# A one-step tRNA-CRISPR system for genome-wide genetic interaction mapping in mammalian cells

**DOI:** 10.1038/s41598-019-51090-3

**Published:** 2019-10-10

**Authors:** Yulei Zhao, Kathrin Tyrishkin, Calvin Sjaarda, Prem Khanal, Jeff Stafford, Michael Rauh, Xudong Liu, Tomas Babak, Xiaolong Yang

**Affiliations:** 1 Department of Pathology and Molecular Medicine, Queen’s University, Kingston, Ontario Canada; 2 Department of Psychiatry, Queen’s University, Kingston, Ontario Canada; 3 Queen’s Genomics Lab at Ongwanada (QGLO), Ongwanada Resource Center, Kingston, Ontario Canada; 4 Center for Advanced Computing, Queen’s University, Kingston, Ontario, Canada; 5 Department of Biology, Queen’s University, Kingston, Ontario Canada; 6 Department of Research Analytics, Celgene Corp, San Francisco, California USA; 7 Present Address: Human Oncology and Pathogenesis Program, Memorial Sloan Kettering Cancer Center, New York, NY USA

**Keywords:** Genetic techniques, Molecular medicine

## Abstract

Mapping genetic interactions in mammalian cells is limited due to technical obstacles. Here we describe a method called TCGI (tRNA-CRISPR for genetic interactions) to generate a high-efficient, barcode-free and scalable pairwise CRISPR libraries in mammalian cells for identifying genetic interactions. We have generated a genome- wide library to identify genes genetically interacting with TAZ in cell viability regulation. Validation of candidate synergistic genes reveals the screening accuracy of 85% and TAZ-MCL1 is characterized as combinational drug targets for non-small cell lung cancer treatments. TCGI has dramatically improved the current methods for mapping genetic interactions and screening drug targets for combinational therapies.

## Introduction

Genetic interaction networks, typically generated through systematic assessment of phenotypic effects linked to combinations of genetic perturbations, are a powerful approach for unbiased mapping of functional dependencies in biological systems^1^. This not only provides potential essential genes as drug targets, but also allows the identification of combinational drug targets, which would help avoid and conquer drug resistances, one of the major obstacles for disease therapy^2^.

Although various screens were performed using RNA interference (RNAi) system to map genetic interactions or networks or combined drug targets in mammalian cells. High off-target effects and low knockout efficiency of RNAi in cells limited its usage^3,4^. The development of CRISPR-Cas9 system, in which a single guide RNA (sgRNA) targets genomic DNA sequences homologous to gRNA in complex with the Cas9 endonuclease^5,6^, is by far the best tool for genetic screen in mammalian system with low off-target effect. However, most of the genome-wide CIRPSR-Cas9 genetic screens were performed by targeting single genes in the human genome^4,7–10^, which is not suitable for systematically mapping genetic interaction networks. Although a couple of genetic interaction screenings using multiplex CRISPR were reported^11–14^, it requires multiple plasmid transfection or cloning steps, large constructs and complex combinations of promoters, which limit its usage in genome-wide genetic screening due to high risk of losing library components during library construction. Besides, despite the use of multiple promoters improves the transcription efficiency of pairwise sgRNAs, it may also cause uneven expression of pairwise sgRNAs in different cells. Additionally, the application of barcode to label multiple gRNAs has been found result in half of the mismatch of gRNA-barcodes due to lentiviral template switching^15^. These two factors would dramatically disturb the final data readout and analysis. Recently, an endogenous tRNA processing system is reported for constructing multiplex CRISPR in different organisms^16–18^, suggesting that tRNA processing system could dramatically enhance the processing efficiency and function of dual sgRNAs transcribed from a single transcript.

The deficiencies of current multiplex CRISPR systems mentioned above have considerably restricted their applications for genetic interaction screens for combinational drug targets, especially large-scale screens such as genome-wide studies. Here we solve all of these issues through a method that is easier to implement. The tRNA processing system allows pairwise sgRNA expression in a single cell and avoids the introduction of the extra promoter and therefore eliminates the uneven expression risk of pairwise gRNAs in different cells, suggesting a potential of this system for genome-scale genetic screen in mammalian cells with multiplex CRISPR. We therefore conceived our TCGI (**t**RNA-**C**RISPR for **g**enetic **i**nteractions) approach by applying this tRNA-processing system to generate a high-efficient, barcode-free and scalable pairwise tRNA-gRNA CRISPR libraries in mammalian cells for identifying genetic interactions and potential combinational drug targets.

As an oncogene and Hippo pathway core component, *TAZ* (also called *WWTR1*) plays a very critical role in malignancies of different cancers^19^, suggesting TAZ as a potential target for cancer treatment. In order to better understand the functions of TAZ and to validate the TCGI approach, we therefore designed and established a pairwise CRISPR library targeting both *TAZ* as well as the whole human genome to study the genome-scale genetic interactions with *TAZ* and to find potential combinational targets with TAZ for cancer treatment.

## Results

### The tRNA-gRNA system works robustly in mammalian cell lines

**Figure 1:**
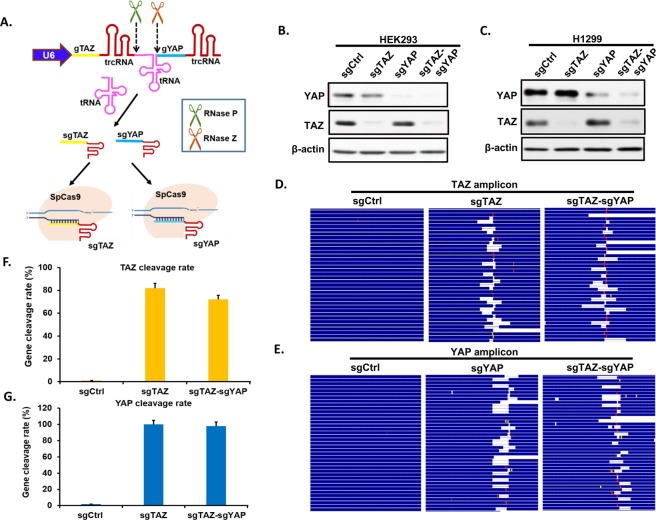
Efficient tRNA-gRNA CRISPR system in mammalian cell lines. (**A**) A schematic showing how tRNA-gRNA system works in mammalian cells. sgRNAs targeting *TAZ* (sgTAZ) and *YAP* (sgYAP) are linked through tRNA to form a single transcript driven by U6 promoter. The tRNA precursor is cut off from the transcript through the endogenous RNase P and RNase Z, during which sgTAZ and sgYAP are separated to form complexes with Cas9 individually to target relative genes with complimentary sequences. See also Fig. S1. (**B**,**C**) HEK293 (**B**) and H1299 (**C**) cells were infected with lentiviral constructs expressing a single sgRNA targeting individual genes (sg*TAZ*, sg*YAP*) or a *tRNA* franked with two *gRNAs* targeting both *TAZ* and *YAP* (sgTAZ-sgYAP). Cell lysates were extracted after 14 days post infection/selection and subjected to western blotting for detection of YAP and TAZ protein expression levels compared with lysates from sgControl (Ctrl) infected cells. β-actin was used as loading control. (**D**–**G**) Genomic DNAs (gDNAs) were extracted from HEK293 infected with lentiviral constructs of single sgTAZ, sgYAP, and double sgTAZ-sgYAP after 14 days culture post infection/selection. TAZ (**D**,**F**) and YAP (**E**,**G**) amplicons containing relative sgRNA targeting regions were amplified and sent for next generation sequencing (NGS). Sequences of relative genes were further mapped to relative gene locus to check the efficiency of both single and double CRISPR system (**D**,**E**). The blank indicates deletions, red bars indicate mutations and yellow bars represent insertions. The cleavage of both TAZ (**F**) and YAP (**G**) genes were counted and cleavage rate was calculated and presented in the charts.

To evaluate the use of a tRNA spacer to efficiently yield functional processed guides in a mammalian system^18^, we tested with sgRNAs targeting *TAZ* and *YAP*. We chose the most effective sgRNAs targeting either *TAZ* or *YAP*, which were confirmed previously in our lab. Then we generated lentiviral constructs expressing a single sgRNA targeting individual gene (i.e. *TAZ*, *YAP*) or a *tRNA* flanked with two *gRNAs* targeting both *TAZ* and *YAP* (Fig. 1A), in which gTAZ was in front of tRNA (gRNA1 position) and gYAP was behind tRNA (gRNA2 position), driven by a single U6 promoter. We infected HEK293 and H1299 with above lentiviruses at MOI = 0.3 and compared the pairwise tRNA-sgRNA with regular single gene-targeting CRISPR system for gene knockout in both cell lines. Fourteen days post lentiviral infection and puromycin selection, similar levels of YAP and TAZ knockdown were observed for both pairwise tRNA-gRNA and single CRISPR systems (Fig. 1B,C). To further confirm the gene modifications, the DNA surrounding the targeted DNA (~100 bp) was later amplified by two rounds of PCR and subjected to next-generation sequencing (NGS) with Illumina Miseq sequencer. Alignment of targeted DNA sequence with corresponding wild-type genomic DNA sequence further confirm that sgTAZ-sgYAP makes indels or cleavages comparable to those mediated by single sgTAZ or sgYAP (Fig. 1D–G). These results suggest that tRNA-sgRNA system functions robustly in mammalian cell lines and the cleavage efficiency of each sgRNA in the same construct is independent of their positions in the transcript.

### Establishment and evaluation of dual gRNA library targeting TAZ-genome through TCGI method

**Figure 2:**
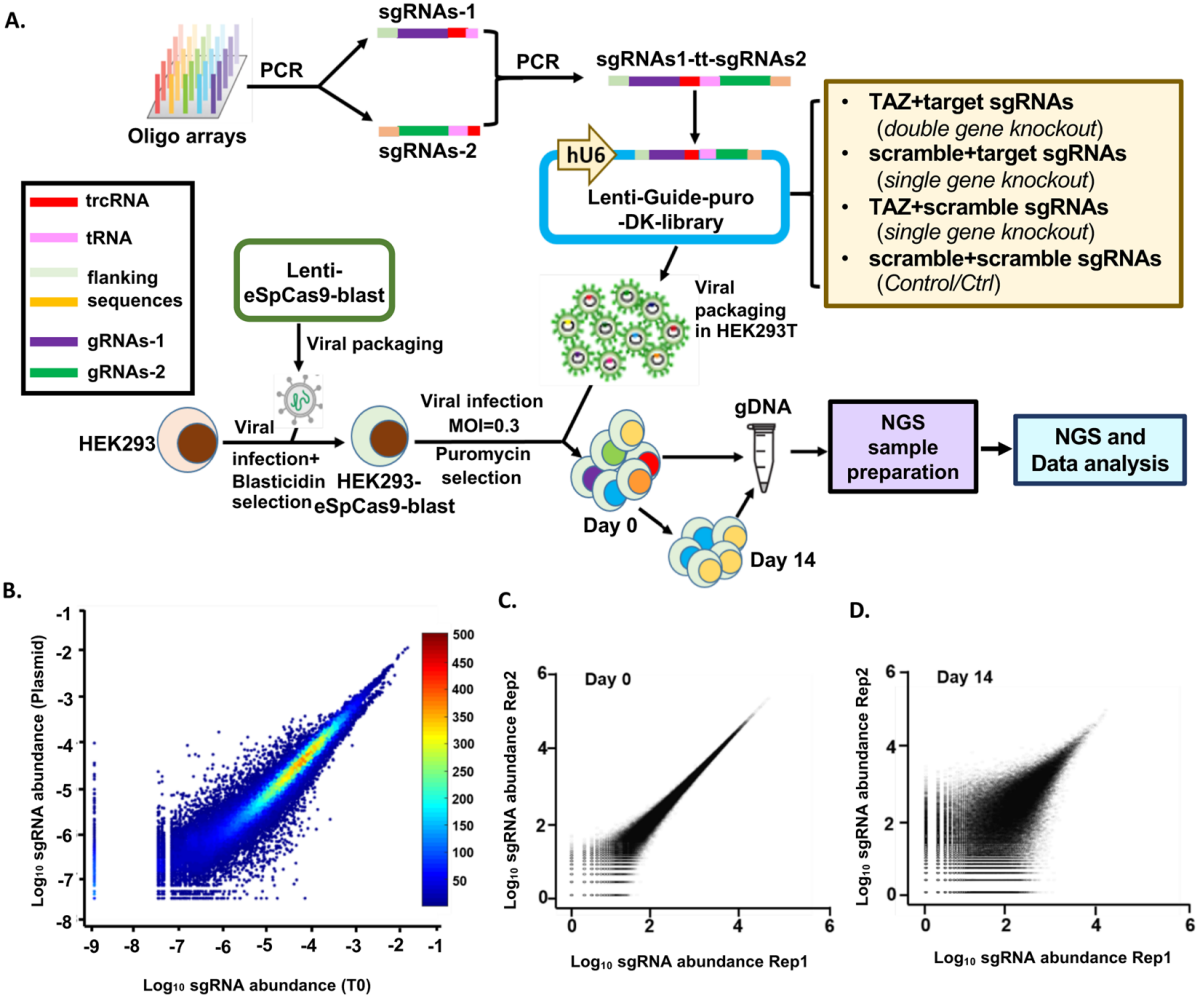
Establishment and evaluation of dual gRNA library targeting *TAZ*-genome through TCGI method. (**A**) A schematic method for establishing TCGI platform for differential growth screen. Array-synthesized oligos were amplified through PCR with relative primers. Then two types of PCR pools were assembled together into full length of segments for cloning into pLenti-Guide vector to generate the lenti-Guide-puro-double knockout (DK) library, including both double gene knockout and single gene knockout as clarified in the figure. HEK293 cells were transduced with lentivirus plasmid containing eSpCas9 and blasticidin resistant gene to generate HEK293-eSpCas9-blast cell line, which stably expresses eSpCas9. Then, the DK library was transduced into HEK293-eSpCas9 cells. After puromycin selection (Day 0), these cells were further cultured for 14 days (Day 14) and gDNA was extracted from both Day0 as well as Day 14 for NGS sample preparation. Deep sequencing data from NGS were analyzed to find out genes synergistically affect cell growth. (**B**) Correlations of double-sgRNA constructs in the DNA library (Plasmid) and after transduction and puromycin selection (T0). Spearman Correlation = 0.977. Color bar shows the density of data. (**C**,**D**) Characterization of biological replicates of the double-sgRNA libraries (Rep1 and Rep2, two biological replicates) at day 0 (**C**) and at day 14 (**D**).

We next applied this pairwise tRNA-gRNA system to genome-wide screen for genes interacting with *TAZ*, an oncogene involved in cell growth regulation in many types of cancers^19–21^. We first used array-based oligonucleotide synthesis to generate the tRNA-gRNA library targeting TAZ and the whole human genome (Fig. 2A). gRNA sequences for over 19,000 genes (3 gRNAs/gene) from the human genome were selected from the database generated by David Root group, in which the gRNAs are optimized with maximum activity and minimum off-target effect^22^. Six hundreds of scrambled nontargeting control sequences (Ctrl) absent from the genome, which accounts for about 1% of the total gRNAs in the pools, were used as negative Ctrl (Table S1). Two pools of oligos containing sgRNA1 plus 5′ end of tRNA sequence and 3′end trcRNA plus tRNA in addition to gRNA2 sequences were first synthesized and amplified by PCR (See Star Methods for more details). Two PCR products were assembled into full length by overlapping PCR (Fig. 2A) and subsequently cloned into Cas9-less pLentiGuide vector. In this way, we generated the dual gRNA library consisting of 231,728 different combinations. In each combination, gRNA1 is designed to target either *TAZ* or one of *Ctrls*, whereas gRNA2 is designed to target any gene in the human genome or *Ctrl* (Fig. 2A). For the screen, we generated HEK293 cells stably expressing engineered SpCas9 (eSpCas9), a mutant Cas9 with extremely low off-target effect^23^. HEK293-eSpCas9 cells were infected in duplicate with the dual-gRNA lentiviral library and selected in puromycin (2 μg/mL) for 4 days. Right after selection, cells were harvested for genomic DNA extraction (set as T0). Similarly, genomic DNA was extracted from cells maintained in exponential growth phase for 14 days (T14). Dual-sgRNA cassettes in the library plasmid and in cells at T0 and T14 were amplified by PCR and the frequency of each dual-gRNA in the library is quantified by deep sequencing.

Our deep sequencing results indicate that there is no significant bias between the gRNA abundance of the library DNA constructed and the one after transduction of library by lentivirus in HEK293-eSpCas9 cells (T0) (Fig. 2B), suggesting that our method to generate and transduce large-scale library to cells for screening is efficient. In addition, the two biological replicates at different time points (T0, T14) were highly reproducible (Fig. 2C,D). Therefore, all of these results show that our screening approach is able to deliver high-reproducible data for differential growth screen.

### Efficacy analysis of High-throughput TAZ-genome genetic interaction screen

**Figure 3:**
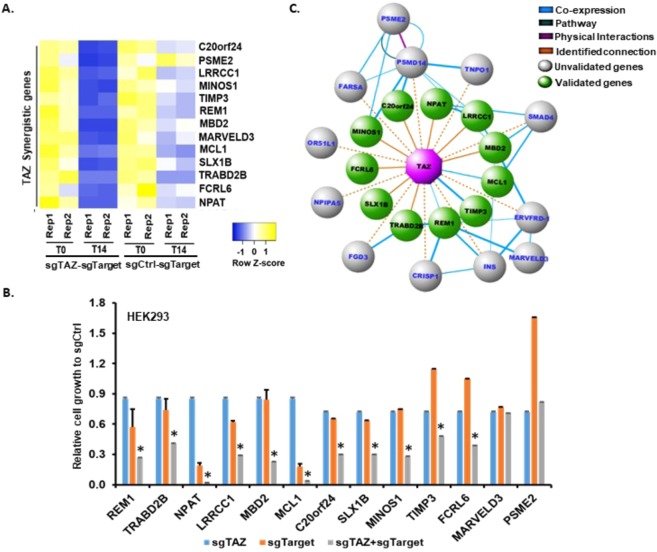
Efficacy analysis of High-throughput TAZ-genome genetic interaction screen. (**A**) Heatmap showing expressions of selected synergistic genes interacting with Control (Ctrl) or TAZ in both T0 and T14 replicated samples (Rep1, Rep2). (**B**) Validation of selected synergistic genes with cell proliferation assay. Selected genes were targeted by relative sgTargets with single CRISPR-Cas9 system in HEK293-sgTAZ or HEK293-sgCtrl cell lines. Cells were plated in triplicate into 24-well plates. The next day (T0) and 14 days (T14) post plating, cells were harvested and cell numbers were counted by Flow Cytometry. The relative fold change between T14 and T0 of each cell line to that of sgCtrl cell line was calculated and presented as mean ± SD (n = 3). “*” represents the differences between cell lines containing sgTAZ + sgTarget and those of either individual sgTAZ or sgTarget were significant with *P* < 0.05. Validation of non-TAZ interactive genes is presented in Fig. S3A. (**C**) Genetic network of genes interacting with TAZ. Top 23 genes with synergistic interaction with TAZ negatively regulating cell growth from the screen were used to generate an interaction network using GeneMania. The final network reflecting 11 validated interactions (green nodes) with TAZ (purple node) were constructed in Cytoscape.

**Figure 4:**
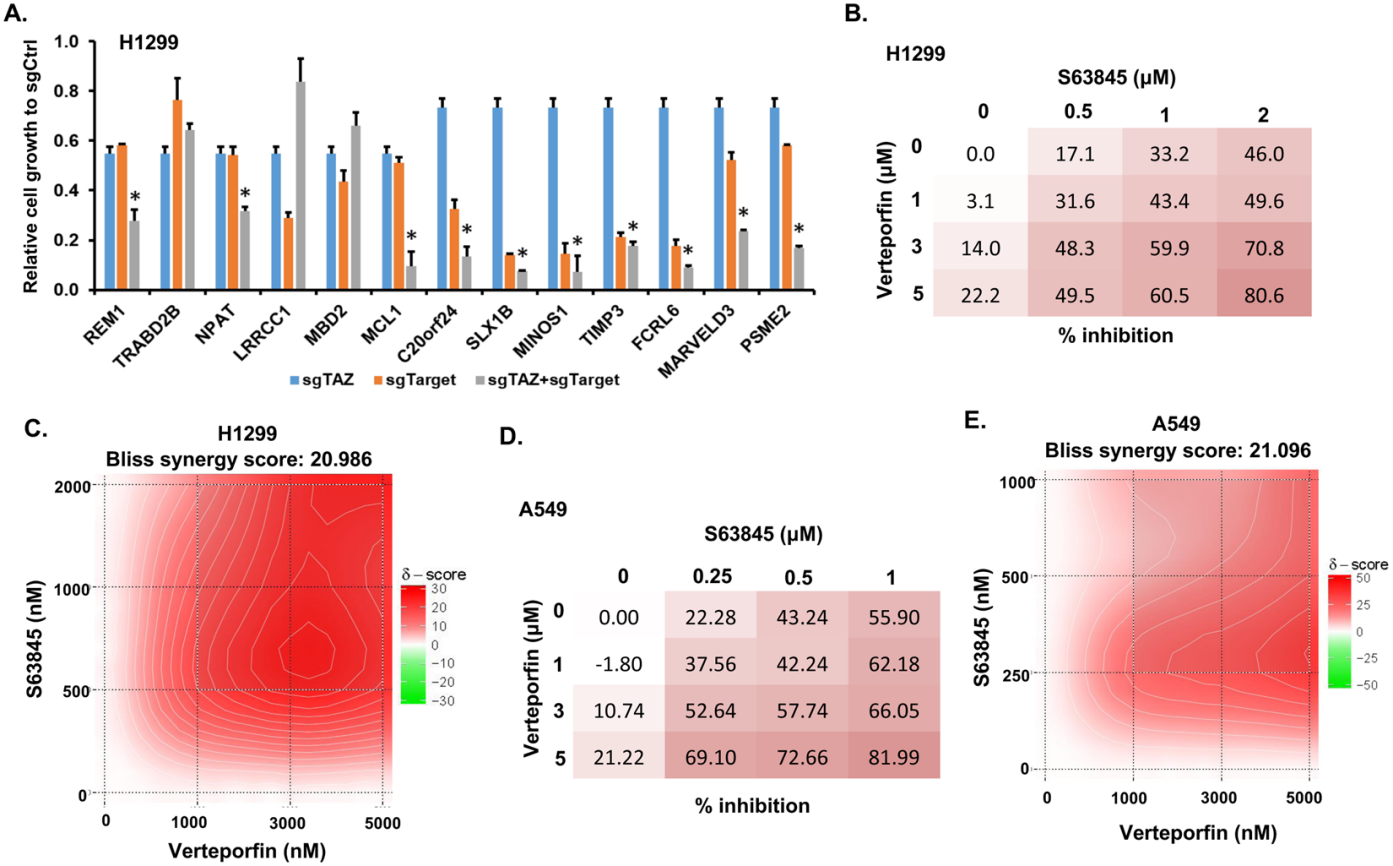
MCL1 and TAZ are potential combinational drug targets for NSCLC treatment. (**A**) Validation of selected synergistic genes with cell proliferation assay in H1299 cells. The relative fold change between T14 and T0 of each cell line to that of sgCtrl cell line was calculated and presented as mean ± SD (n = 3). “*” represents the differences between cells expressing sgTAZ + sgTarget and those expressing individual sgTAZ or sgTarget were significant with P < 0.05. See also Fig. S3B for validation of non-TAZ interactive genes. (**B**) Validation of MCL1 and TAZ interaction in H1299 cells with inhibitors. H1299 cells were treated with MCL1 inhibitor S63845 alone or together with Verteporfin to target TAZ at relative concentrations mentioned in the figure for 3 days. Then cell viability was measured through CellTiter-Glo (Promega), the rate of inhibition or viability was further calculated based on the measured values of control (DMSO) and those treated with drugs. (**C**) A 2D plot of Bliss synergy score was generated with SynergyFinder showing that the two inhibitors used in (**B**) has synergistic effect. (**D**) Validation of MCL1 and TAZ interaction in A549 cells with inhibitors. The treatment was similar as described in (**B**). E. Bliss synergy score of treatment performed in (**D**).

For data analysis, the noises resulting from sequencing were first eliminated by normalizing data with gCtrl-gCtrl combination reads (Fig. S2). Then, the dual-gRNAs with less than 5 counts per million in less than 2 samples were filtered out to avoid the noises caused by low-sequencing depth samples. We identified a set of gene perturbations affecting cell viability through comparing gRNA depletions in T14 with those in T0. Further genes showing synergistic effect with TAZ were listed from comparison of their fold changes with those of scramble-gene and gTAZ-gCtrl combinations (Table S2). Among these genes, 231 of them have previously been shown to correlate with TAZ (e.g., coexpression through either DNA or RNA microarray analyses or protein interactions via Mass spectrometry detection, etc) (Table S2). To further examine the efficiency of this TCGI platform, we chose 13 candidate genes synthetically interacting with TAZ (Table S2, Fig. 3A) and validated them individually by CRSIPR-Cas9 in HEK293-sgCtrl and HEK293-sgTAZ cell lines. Cell proliferation of HEK293-sgTAZ, sgTarget, and sgTAZ-sgTarget cells was compared with that of HEK293-sgCtrl. Among these 13 genes, 11 have synergistic effect with TAZ on cell proliferation (Fig. 3B), suggesting that our genome-wide TCGI screen has an accuracy of 84.6%. In contrast, 7 genes selected with no TAZ interactions does not show any synergistic effect with TAZ (Fig. S3A), further suggesting that our TCGI screen is very specific. We also generated a genetic network of TAZ interacting genes with our analysis data as well as published literature (Fig. 3C), from which we could have a better idea on the interactions among those genes. Additionally, we also validated these genes separately in a non-small cell lung cancer (NSCLC) cell line H1299 (Figs 4A and S3B). Significantly, 8 out of 11 TAZ-interacting genes identified in HEK293 were also confirmed in H1299 cells (Fig. 4A), suggesting that the TCGI system can be applied to different cell lines.

### MCL1 and TAZ are potential combinational drug targets for NSCLC treatment

In addition to study the genetic interactions with TAZ, we further examined the potential application of our approach in identifications of combinational drug targets. Among the validated genes, we only found that MCL1 has commercial inhibitors currently. Since dysregulation of TAZ is involved in NSCLC^24^, we tested whether combined treatment of NSCLC with inhibitors of MCL1 and TAZ are beneficial for therapy. We therefore treated H1299 lung cancer cells with MCL1 inhibitor S63845^25^ individually or together with Verteporfin that inhibits TAZ function^21,26^ to further validate our interaction result as well as test if there are synergistic inhibition effects in H1299 cell growth. While individually MCL1 or TAZ inhibitor did not show significant effect on cell inhibition, the combination treatment dramatically inhibits the cell proliferation (Fig. 4B). The Bliss independence model analysis^27^ indicates the two inhibitors showing synergistic effect in H1299 cells (Fig. 4C). This synergistic effect was also confirmed in another NSCLC cell line A549 (Fig. 4D,E) as well as using another MCL1 specific inhibitor, A1210477 (Fig. S4A,B), suggesting a specific on-target effect of the treatment. All these results not only confirmed our functional genetic screen results, but also suggest that MCL1 and TAZ could be new combinational targets for clinical NSCLC treatment.

## Discussions

Mapping the genetic network of certain genes or pathways is an efficient way to comprehensively understand their biological functions in different conditions^1,4,28–31^. It also helps to find the most effective targets for new drug development and/or for combinational therapy. Although genome-wide genetic interaction screens have been successfully performed in yeast^29,31^, not many labs so far are able to perform genome-wide genetic interaction screens in mammalian cells due to technical limitations.

TAZ is a transcriptional co-activator that is a major player of the Hippo pathway^32–35^. It is involved in the development and progression of various types of cancers^19–21^, suggesting that TAZ could be a potential drug target for cancer treatment. Some studies also indicate additional regulations of TAZ^36–40^. However, so far there is no study for a comprehensive understanding of TAZ genetic interactions.

Here we have established a simple and high-efficient TCGI system that can be used to perform genome-wide pairwise CRISPR genetic screen in mammalian cells. By using this system, we generated a library containing over 230,000 pairwise gRNAs to identify and validate many cellular genes functionally interacting with TAZ in regulating cell proliferation. Functional annotations of identified genes performed with DAVID bioinformatics resources^41^ not only confirm the function of TAZ in transcription regulation, but also suggests potential novel roles of TAZ in lipid metabolism, Neurodegeneration, electron transport, etc (Table S3), which provides useful information for future TAZ studies. More significantly, we identified and confirmed MCL1 as a combinational therapy target with TAZ for inhibiting NSCLC cell growth. This not only provides useful information for effective NSCLC treatment, but also indicates that our system is suitable for screening combinations of existing drug targets for generating new therapeutic strategies or identifying potential novel combinatorial targets for drug development. In future, more work needs to be done to clarify the mechanism of the synergistic effect by targeting both MCL1 and TAZ in cell lines and animal models *in vivo*.

Since we started with whole-genome screen rather than a specific screen of the genes with existing drugs to target. Therefore, most of the genes we identified from the screen have no commercial drugs to target, which limited our validation method by using drugs/inhibitors treatments. However, by constructing a library specific targeting druggable genes, our method could be easily adapted to screen targets for combinational therapies.

In our method, the pairwise gRNAs are expressed from one transcript, which is driven by a single promoter and cleaved efficiently by endogenous tRNA processing machinery in a single cell. This allows two gRNAs to target two genes with equal knockout efficiency. Moreover, the single cloning step allows more flexible and scalable constructions for various library sizes or gene groups (e.g. kinome, phosphatase, druggable genes). Although in the present screen, only single TAZ-genome library was performed with TCGI, another library for studying the Hippo pathway and whole genome interactions could also be constructed with TCGI (data not shown in this paper) robustly. Despite the two sgRNAs in one vector share same trcRNA sequences in our method, resulting in two products (sgRNA1 only and sgRNA1-tRNA-sgRNA2) during NGS sample preparation, the sizes of the two products are quite different, which could allow a gel purification of desired products based on size differences. In future, using different trcRNA sequences for different gRNAs in the construct would be helpful to avoid the double products. Overall, the TCGI system is an easy-performable method allowing studies of genetic interactions in mammalian cell lines to identify potential targets for combinational therapies.

## Methods

### Cell culture

HEK293 and HEK293T cells were cultured in Dulbecco’s Modified Eagle complete medium (DMEM) (Sigma-Aldrich) supplemented with 10% heat-inactivated fetal bovine serum (FBS) (Sigma-Aldrich) and 1% penicillin/streptomycin (P/S). H1299 lung cancer cell line was maintained in Roswell Park Memorial Institute (RPMI)−1640 medium with 10% FBS and 1% P/S. All cell lines were incubated at 37 °C, 5% CO_2_ and passed every 3–4 days.

### Establishment of tRNA-gRNA-dual targeting system through a two-step cloning

To check the tRNA-sgRNA system works in mammalian cells or not, the establishment of double-gRNA-targeting TAZ-YAP was performed through a two-step cloning process (Fig. S1). A synthesized two gRNAs (gTAZ-gYAP) connected with a BsmBI cutting sequence was first cloned into a BsmBI-digested LentiCRISPR-v2 backbone [a gift from Feng Zhang (Addgene plasmid # 52961)] containing a polymerase III promoter (h*U6*) and *SpCas9*. Further digested with BsmBI, a synthesized trcRNA-tRNA (scaffold sequence-tRNA sequence) oligo was cloned in between the two gRNAs in the recombined LentiCRISPR-v2 plasmid to generate the sgRNA1-tRNA-sgRNA2-CRISPR construct, which is the double-gRNA-targeting construct.

### One-step cloning method generating dual-targeting CRISPR libraries and differential growth screen

The two-step cloning method costs extra money and labor work, with high risks of losing elements for producing library pools. For genome-wide pairwise tRNA-gRNA library construction, we conceived a one-step method with the combination of PCR and array-based oligonucleotides synthesis to establish the dual-targeting CRISPR libraries as followings.

### Design of gene constructs

The gRNAs targeting TAZ (WWTR1) and all the genes from human genome were selected from dataset generated by David Root Group in 2016, in which the gRNAs were optimized with highly active on-target effect^22^. Three gTAZ and one scramble non-targeting sequence as negative control (gCtrl) were included in sgRNA1 position of the library and gRNAs targeting human genome together with 600 scramble non-targeting sequences as negative controls (Ctrl) were designed in sgRNA2 position. Therefore, the final library would contain 231,728 constructs, including both *TAZ*-genome-knockout and single-gene-knockout constructs (Fig. 2A). Two pools of oligos were synthesized by CustomArray as BsmBI-gRNA1-trcRNA-tRNA and trcRNA-tRNA-gRNA2-BsmBI with each length of 129 bp (for detail sequences please refer to Table S1).

### Library Construction and cloning

The array-based customized oligonucleotides (BsmBI-gRNA1-trcRNA-tRNA; trcRNA-tRNA-gRNA2-BsmBI, Table S1) were amplified out of the synthesized pool by PCR using PrimeStar (Takara) with specific primers as followings,

For “BsmBI-gRNA1-trcRNA-tRNA” oligos (129 bp):

BsmBI-F: CAGATGACTCGTCTCGCACC

trc-tRNA-R: CTGGTGCTTTGTTGCACCGA

For “trcRNA-tRNA-gRNA2-BsmBI” oligos (129 bp):

trc-tRNA-F: CCGAGTCGGTGCAACAAAGC

BsmBI-R: GGGCCCTTTCGTCTCCAAAC

Next, the full-length segments (BsmBI-gRNA1-trcRNA-tRNA-gRNA2-BsmBI, 227 bp) were generated with above amplified oligos through overlapping PCRs. The pool of PCR products was purified with PCR purification kit (Qiagen). Then these purified pools of PCR products were digested with BsmBI (Fermentas) and ligated with T7 ligase into the LentiGuide-Puro vector [a gift from Feng Zhang (Addgene plasmid#52963)] at the ratio of insert:vector = 1:6. After purification with QIAquick Nucleotide Removal kit (Qiagen), 2 μL of the purified ligation products was transformed into 25 μL of ElectroMAX Stbl4 Competent Cells (Thermo Fisher Scientific) through electroporation according to the manufacturer’s protocol. To ensure no loss of representation, 40 parallel transformations were performed using the same ligation reaction and plated onto 60 × 150 mm plates with ampicillin selection (50 μg/mL), which yielded 250 X library coverage. Colonies were scraped off plates and combined before plasmid DNA extraction using Endotoxin-Free Plasmid Maxiprep (Qiagen).

### Library lentivirus production and purification

To produce lentivirus, HEK293T cells were transfected with library DNA with PolyJet (SignaGen) according to the manufactory’s instructions. After 24 hours, the media was changed to complete DMEM (with 10% FBS and 1% P/S) containing 10 mM sodium butyrate (Bioshop). After another 24 hours incubation, the media was collected and centrifuged at 500 g at 4 °C for 10 min to pellet cell debris. The supernatant was collected and mixed with 1/3 volume of Lenti-X^TM^ Concentrator (Clontech). Then the mixtures were incubated at 4 °C for overnight before being spun down at 1,500 g at 4 °C for 45 min. The virus pellets were resuspended with cold 1×PBS at 1/100 dilution and aliquoted before being frozen by liquid nitrogen.

### Establishment of HEK293-eSpCas9-blast cells

Lentivirus of eSpCas9-blast was produced as above and transduced into HEK293 cells at MOI = 1. The cells were grown in complete DMEM containing blasticidin (10 μg/mL) to select cells with integrated eSpCas9. Nearly 100% killing was observed in cells without the Cas9 vector after 5 days of exposure. These cells were maintained in complete DMEM and passed every three days.

### Titration of the library virus

To find optimal virus volumes for achieving MOI of 0.3, HEK293-eSpCas9 cells were plated into a 12 well plate in complete DMEM. The next day, one well of cells was counted and the rest wells were infected at different amount of library viruses together with a no-virus control. Each virus was infected into two wells of cells. The next day, one well of same virus infected cells were treated with puromycin at 2 μg/mL. After 3 days, cells were counted to calculate a percent transduction. Percent transduction is calculated as the following:

$$ \% \,{\rm{transduction}}={\rm{Cell}}\,{\rm{number}}(+{\rm{puromycin}})/{\rm{Cell}}\,{\rm{Number}}(-{\rm{puromycin}})\times 100.$$

### Differential growth screen

HEK293-eSpCas9 cells were infected with the library virus at MOI of 0.3 and coverage of 250- fold to ensure each cell had zero or one double-gRNA construct. Puromycin (2 μg/mL) selection was started 1 day after transduction, and lasted for 4 days. Then the selection pressure was maintained with puromycin dose of 0.5 μg/mL throughout the course of the experiment to eliminate cells without gRNAs. After transduction, cells containing integrated gRNAs were maintained in exponential growth phase by harvesting and removing a fraction of the cells approximately every 2–4 days. Genomic DNA (gDNA) was extracted from cells harvested at 0- and 14-day after selection with a Blood and Cell Culture DNA Mini Kit (Qiagen) according to the manufacturer’s protocols. The screening was repeated as two biological replicates.

### NGS library preparation

The genomic DNA samples extracted from above screening were used for generation NGS samples. The double-gRNA cassette was amplified and prepared for deep sequencing through two steps of PCR. The first step was performed with 3 μg input gDNA per 50 μl reaction (60 μg for each sample) with 0.75 μl of PrimeStar polymerase per reaction. The PCR primers were as follows:

NGS_P1-lenti-Guide_F, ACACTCTTTCCCTACACGACGCTCTTCCGATCTATCTTGTGGAAAGGACGAAA

NGS_P2-lenti-Guide_R, GTGACTGGAGTTCAGACGTGTGCTCTTCCGATCTGCTATTTCTAGCTCTAAAAC

The thermocycling parameters were: 98 °C for 10 s; 55 °C for 15 s and 68 °C for 10 s, in total set 21 cycles. The numbers of cycles were tested to ensure that they fell within the linear phase of amplification. Amplicons (300 bp) for each sample were pooled and the second step of PCR was performed with 3 × 100 μL per reaction containing 5 μl of pooled 1^st^ PCR amplicons to attach Illumina adaptors and indexes. The thermocycling parameters were: 98 °C for 10 s; 60 °C for 15 s and 68 °C for 10 s, in total set 14 cycles. The pooled amplicons were size-selected and purified on 2% agarose gel. The purified products were quantified by Qubit (Thermo Fisher) and mixed in equal amount before being sent for pair-end Illumina NextSeq sequencing at the Lunenfeld-Tanenbaum Research Institute, Sinai Health System in Toronto.

### Processing of paired-end reads

Data preprocessing and sequence alignment was performed using custom Python and R scripts. The quality control of the FASTQ files were performed using FastQC (https://www.bioinformatics.babraham.ac.uk/projects/fastqc/) and MultiQC^42^ software. Raw sgRNA reads were extracted from FASTQ files and were trimmed of scaffold sequences with seqtk (https://github.com/lh3/seqtk). The remaining reads were truncated to 19 bases from the appropriate end and reverse reads were reverse-complemented and assembled as pairs. The resulting pairs were aligned against library gRNA sequences (both gRNA1 and gRNA2). The matched read pairs were further aggregated to compute the total counts for that construct in the relevant sample, which was used for subsequent analysis.

### Data analysis

The background correction was performed by subtracting the median counts of double gRNAs targeting nontargeting controls (gCtrl-gCtrl) for each sample separately. Subsequently, the gCtrl-gCtrl pairs were removed from the data for further processing. The data was then normalized using the trimmed mean of M values (TMM) normalization^43^ (Fig. S2). The differential abundance calls between each pair of gRNAs on Day 14 (T14) and Day 0 (T0) were generated using the EdgeR package^44,45^ in R (version 3.4.4). The phenotypic readout was reflected by logFC results of the differential analysis. Only differentially expressed genes with FDR <0.1 were considered for further analysis.

To determine genes interacting with TAZ to impair cell growth, the average phenotypic readout for same-gene multiples was computed for gCtrl-gTargets and double gTAZ-gTargets. Then, average phenotypic readout of gTAZ-gCtrl was calculated and used to adjust the phenotypic readout of double gTAZ-gTarget to filter out TAZ single effect, which resulted in a set of gTAZ-gTargets. Finally, the ratio between the gCtrl-gTargets and the adjusted gTAZ-gTargets was computed and genes with the ratio <0.5 were considered as TAZ interacting genes in negative regulation of cell growth.

Top 23 of TAZ interactive genes achieved from above analysis were used to generate an interaction network using GeneMania^46^ (version 3.5). The final network (Fig. 3C) that reflects 11 validated interactions was constructed in Cytoscape^47^ (version 3.6.1).

### Correlations

To assess the variance between the two biological replicates, standard Pearson correlation was used to compare the log_10_ read count of each sgRNA, which was plotted using ggplot2 package in R (Fig. 2C,D). Correlation plot showing the log_10_ normalized total counts for T0 vs Plasmid (Fig. 2B) was generated by using MATLAB (Mathworks, Inc., MA, USA, v2016b). Spearman correlation was computed between the normalized total counts for T0 and Plasmid.

### Validation by normal CRISPR

To validate the candidate genes, we first constructed CRISPR-Cas9 recombinant plasmid containing Hygromycin B resistant gene and sgTAZ. We then generated HEK293 and H1299 cell lines transduced with the lentivirus of sgTAZ or sgCtrl to stably knock out TAZ.

We selected 13 genes genetically interacting with TAZ and 7 genes without a synthetic effect with TAZ. We used gRNA sequences from the ones used in the screening library. After cloning these gRNAs into Lenti-CRISPR-v2 plasmid, we produced lentiviruses of these 20 different constructs individually. Then, we generated stable cell lines to target each selected candidate gene (sgTarget) in both sgCtrl cell line and sgTAZ cell line. Further these cell lines together with sgCtrl and sgTAZ cell lines were plated in triplicate into 24 well-plates for cell proliferation assay. The next day (T0) and 14 days (T14) after plating, relative wells of cells were collected and counted with Flow Cytometry. The cell numbers of T14 were normalized with those of T0 and further the fold change relative to sgCtrl were calculated.

### Validation of MCL1 and TAZ interaction with inhibitor treatment

H1299 and A549 cells were plated into 96 well-plate with 10^4^ cells per well. Cells were treated with MCL1 inhibitor (S63845 or A1210477) alone or together with Verteporfin to target TAZ at related concentrations (See Figs 4 and S4) for 3 days. DMSO was used as vehicle control. Then cell viability was measured with CellTiter-Glo (Promega) kit and GloMax® Navigator Microplate Luminometer (Promega) according to manufacturer’s instruction. Cell inhibition or viability ratio was further calculated based on the viability of cells treated with DMSO and those with drugs.

## Supplementary information

Supplementary information

## Data Availability

All raw data could be found in the supplementary; The code generated and used during the current study are available from the corresponding author on reasonable request.
