# Author Correction: A one-step tRNA-CRISPR system for genome-wide genetic interaction mapping in mammalian cells

**DOI:** 10.1038/s41598-021-92514-3

**Published:** 2021-07-08

**Authors:** Yulei Zhao, Kathrin Tyrishkin, Calvin Sjaarda, Prem Khanal, Jeff Stafford, Michael Rauh, Xudong Liu, Tomas Babak, Xiaolong Yang

**Affiliations:** 1 Department of Pathology and Molecular Medicine, Queen’s University, Kingston, ON Canada; 2 Department of Psychiatry, Queen’s University, Kingston, ON Canada; 3 Queen’s Genomics Lab at Ongwanada (QGLO), Ongwanada Resource Center, Kingston, ON Canada; 4 Center for Advanced Computing, Queen’s University, Kingston, ON Canada; 5 Department of Biology, Queen’s University, Kingston, ON Canada; 6 Department of Research Analytics, Celgene Corp, San Francisco, CA USA; 7 Present Address: Human Oncology and Pathogenesis Program, Memorial Sloan Kettering Cancer Center, New York, NY USA

Correction to: *Scientific Reports* https://doi.org/10.1038/s41598-019-51090-3, published online 10 October 2019

The original version of this Article omitted Supplementary Table S1, Supplementary Table S2, and Supplementary Table S3.

These Supplementary Information files now accompany the original Article.

