# Supplementary material for: A one-step tRNA-CRISPR system for genome-wide genetic interaction mapping in mammalian cells
Source: Sci Rep. 2019 Oct 10;9:14499. doi: 10.1038/s41598-019-51090-3 (PMC6787096; doi:10.1038/s41598-019-51090-3)

A one-step tRNA-CRISPR system for genome-wide genetic interaction mapping in mammalian cells

Yulei Zhao[1], Kathrin Tyrishkin[1,#], Calvin Sjaarda[2,3,#], Prem Khanal[1], Jeff Stafford[4], Michael Rauh[1], Xudong Liu[2,3], Tomas Babak[5,6,*], Xiaolong Yang[1,*]

1. Department of Pathology and Molecular Medicine, Queen's University, Kingston, Ontario, Canada
2. Department of Psychiatry, Queen's University, Kingston, Ontario, Canada
3. Queen's Genomics Lab at Ongwanada (QGLO), Ongwanada Resource Center, Kingston, Ontario, Canada
4. Center for Advanced Computing, Queen's University
5. Department of Biology, Queen's University, Kingston, Ontario, Canada
6. Department of Research Analytics, Celgene Corp, San Francisco, California, USA

#   Equal contribution

Running title: One-step CRISPR system for genetic interaction mapping

* Corresponding author: yangx@queensu; tomas.babak@queensu.ca

Keywords: pairwise CRISPR, tRNA, genome-wide, synthetic lethal screen

Supplementary Figures

Supplementary Figure 1. Schematic of two-step cloning method to establish double-targeting CRISPR gRNA1 and gRNA2 were separated with BsmBI cutting site and synthesized into one oligo to be cloned into BsmBI digested lentiCRISPR-v2 vector. Further gRNA1 and gRNA2 in plasmids were separated by BsmBI digestion. Oligo synthesized containing trcRNA and tRNA was further cloned in between the two gRNAs to form the double-targeting CRIPSR construct.

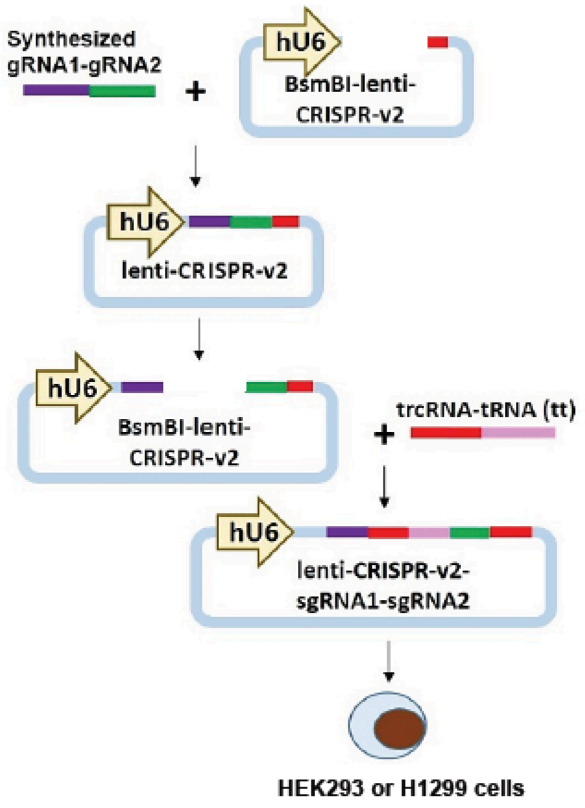

Supplementary Figure 2. Data normalization. The background correction was performed by subtracting the median counts of double gRNAs targeting nontargeting controls (gCtrl-gCtrl) for each sample separately.

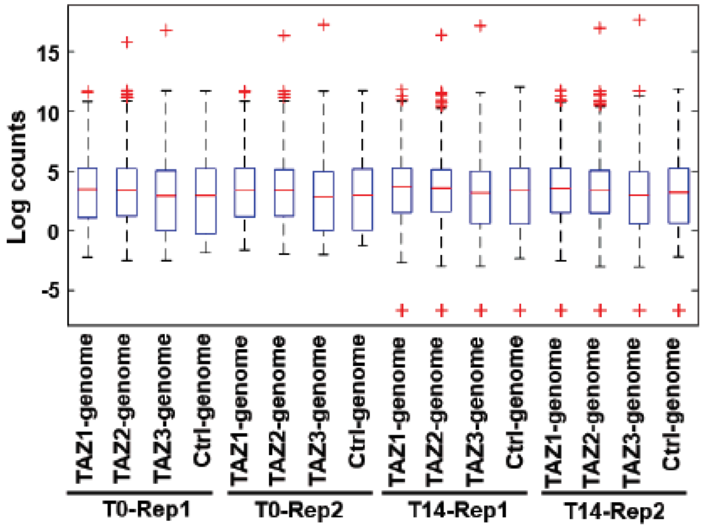

Supplementary Figure 3. Validation of selected no-synergistic genes with cell proliferation assayA-B. Selected 7 genes were targeted by relative sgRNAs with single CRISPR-Cas9 system in HEK293/H1299-sgTAZ or HEK293/H1299-sgCtrl cell lines. Cells were plated in triplicate into 24-well plates. The next day (T0) and 14 days (T14) post plating, cells were harvested and cell numbers were counted by Flow Cytometry. The relative fold change between T14 and T0 of each cell line to that of sgCtrl cell line was calculated and presented as mean ±SD (n=3).

A.

B.

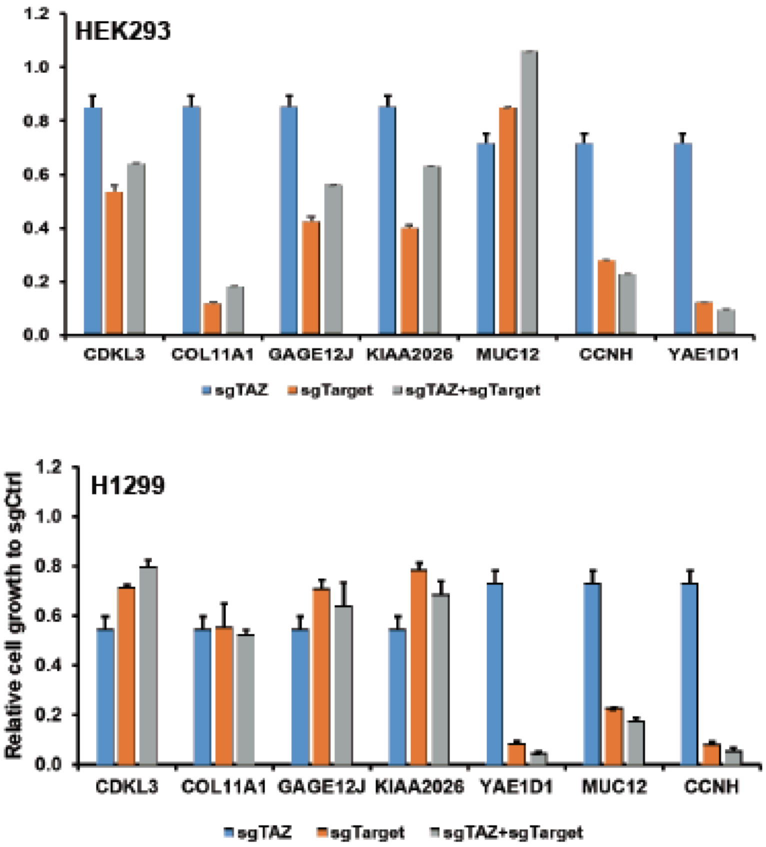

Supplementary Figure S4. Validation of MCL1 and TAZ interactions with inhibitors

A-B. H1299 or A549 cells were treated with MCL1 inhibitor S63845 alone or together with Verteporfin to target TAZ at relative concentrations mentioned in the figure for 3 days. Then cell viability was measured through CellTiter-Glo (Promega), the rate of inhibition or viability was further calculated based on the measured values of control (DMSO) and those treated with drugs. Results were presented as mean ± SD (n=3). "*" represents the differences between cells treated with DMSO and combinational drugs are significant with P < 0.05.

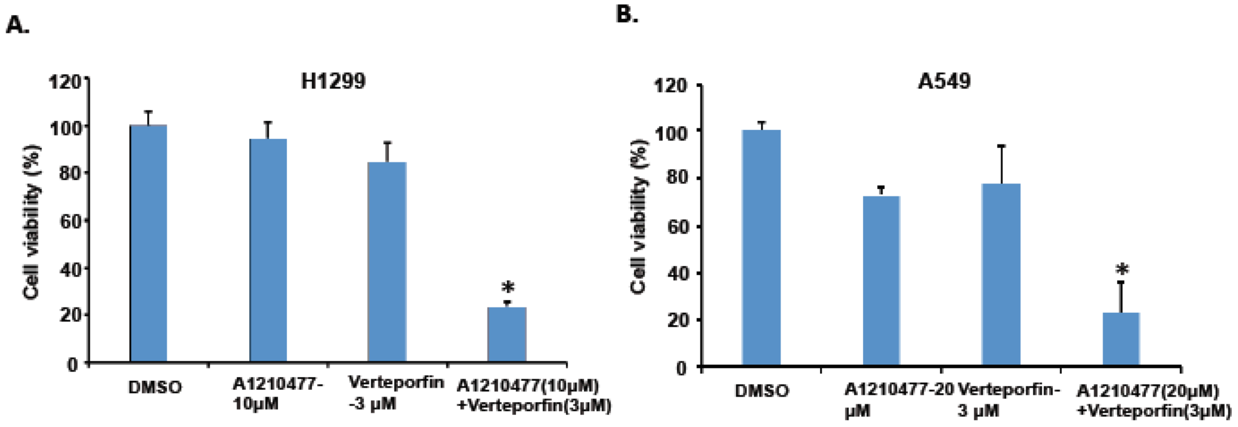

Supplementary Figure 5. Full blots of cropped blots used in Figure 1B & 1C. Parts of the blots used in figure are highlighted in square.

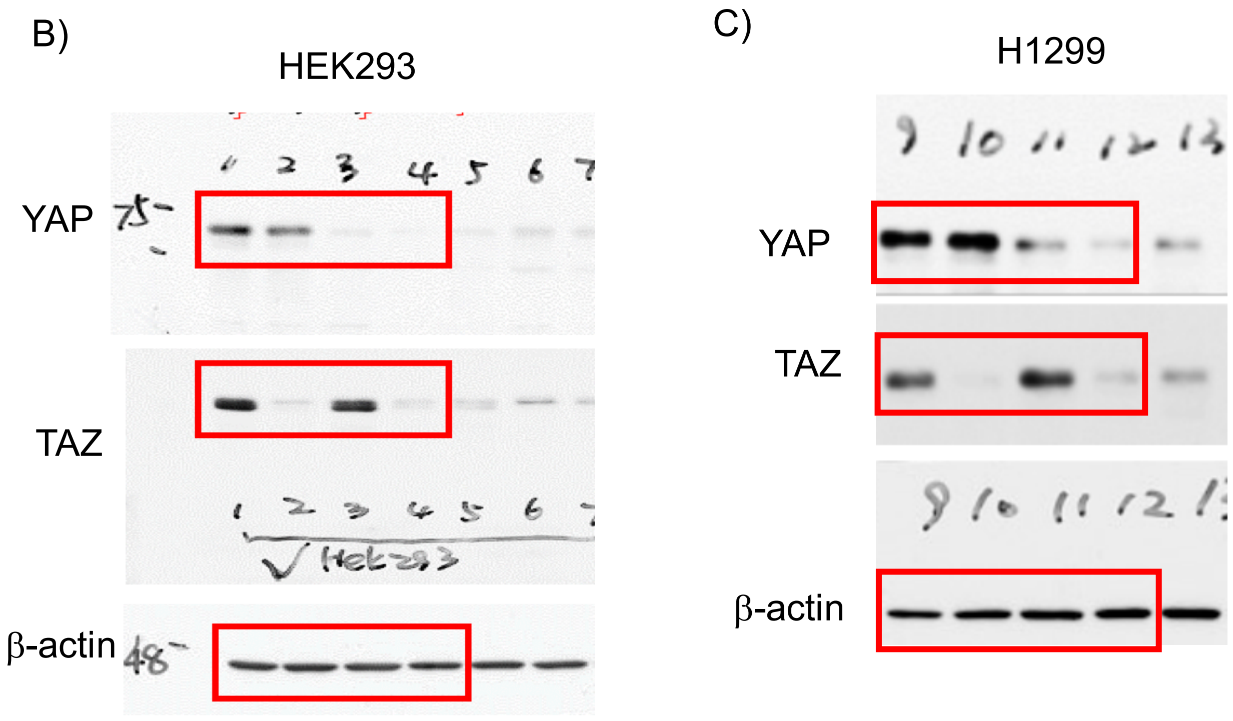

Supplement: Supplementary file 1 — Supplementary information [file 41598_2019_51090_MOESM1_ESM.pdf]
